# Commentary: Embodied Medicine: Mens Sana in Corpore Virtuale Sano

**DOI:** 10.3389/fnhum.2017.00381

**Published:** 2017-07-21

**Authors:** Francesca Pistoia, Antonio Carolei, Simona Sacco, Marco Sarà

**Affiliations:** ^1^Department of Biotechnological and Applied Clinical Sciences, Neurological Institute, University of L'Aquila L'Aquila, Italy; ^2^Post-Coma Intensive Rehabilitation Care Unit, San Raffaele Hospital Cassino, Italy

**Keywords:** body matrix, locked-in syndrome, body representations, virtual reality, motor imagery

We read with interest the recently published paper about the potential role of “*embodied medicine*” (Riva et al., 2017). Authors suggest the use of advanced technologies for altering the experience of being in a body, with the goal of improving the well-being of patients. This paradigm is intriguingly summarized through the key message “*Mens Sana in Corpore Virtuale Sano*” and is recommended for patients with different neurological and psychiatric disorders including neglect, chronic pain, schizophrenia, depression and eating disorders. Here we report about a neurological syndrome which, in our opinion, might greatly benefit from the proposed approach and from simulation/stimulation technologies able to modulate the inner body dimension. This is the *Locked-in Syndrome* (LIS) characterized by a condition of severe motor entrapment due to the interruption of corticospinal, corticobulbar and cortico-cerebellar pathways as a result of a ventral brainstem lesion (Figures 1A,B). Patients are completely entrapped within their body because of quadriplegia, anarthria and lower cranial nerve paralysis, and communicate with the environment only through vertical eye movements and blinking which are the only motor outputs preserved. Despite this, consciousness and sensory pathways (exteroception, proprioception, vestibular inputs, and interoception) are completely conserved. Although cognition is also traditionally considered unaffected, due to the preservation of supratentorial structures, we recently described some non-motor symptoms in these patients, including motor imagery defects, selective emotional dysfunctions and pathological laughter and crying, and interpreted them as a consequence of a body representation disorder (Conson et al., 2008; Sacco et al., 2008; Pistoia et al., 2010). This fits with later volumetric data obtained in these patients, revealing the presence of an unexpected cortical loss involving areas typically associated with the mirror neuron system and the body matrix (Pistoia et al., 2016). As reminded by the authors, an accurate body representation is the result of the effective integration of multisensory (somatosensory, visual, auditory, vestibular, visceral) and motor signals, which provides an evolutionary advantage by maintaining a homeostatic protective *milieu* for human beings. This system, subserved by cortico-ponto-cerebellar pathways, matches bodily sensations and motor intentions in order to protect the body by triggering perceptual and behavioral programs (effectors) when something alters the body and the space around it (Riva et al., 2017). In patients with LIS, the lack of functioning efferent pathways, both at corticospinal and cortico-cerebellar level, may interfere with the body representation system, weaken the boundaries of the body and lead to unexpected symptoms in cognitive domains. Specifically patients are less accurate than healthy control subjects in recognizing others' negative facial expressions, thus confirming that voluntary activation of mimicry is a high-level simulation mechanism crucially involved in explicit attribution of emotions (Pistoia et al., 2010). Similarly, patients with LIS show motor imagery defects including difficulties in mentally manipulating the hands thus endorsing the view that motor imagery is subserved by activation of motor information (Conson et al., 2008). Finally, they can suffer from a pathological laughter and crying syndrome, as a result of a continuous disagreement between preserved centripetal bodily sensations and affected centrifugal motor outputs (Sacco et al., 2008). All these symptoms may be interpreted as the result of a body matrix disorder (Conson et al., 2009, 2010; Babiloni et al., 2010; Pistoia et al., 2016).

**Figure 1:**
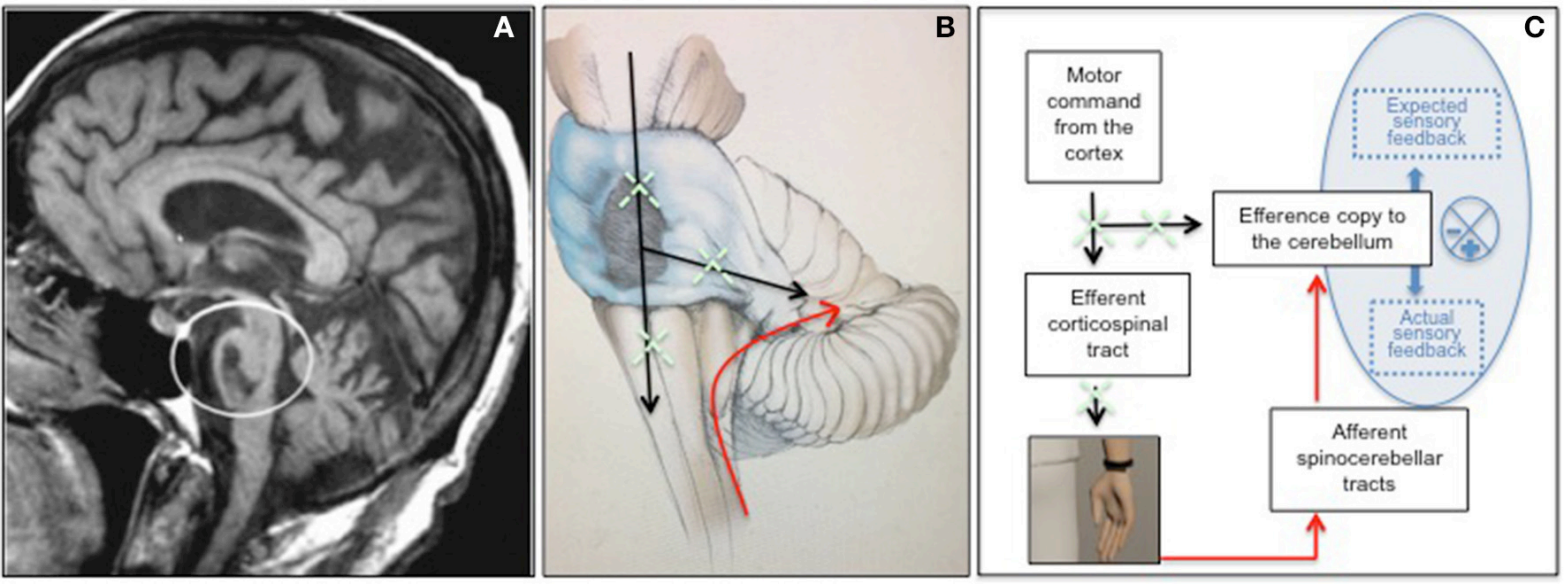
Ventral brainstem lesion in the locked-in syndrome as shown by MRI **(A)**; graphical representation of interrupted efferent corticospinal and corticocerebellar tracts (in black) and preserved afferent spinocerebellar tracts (in red) in LIS patients **(B)**; hypothesized mechanism by which Sonoception technology may contribute to reduce the mismatch between the efferent (defective) and the afferent (healthy) pathways in LIS and to restore properly working forward programs **(C)**.

To date, when reasoning about embodiment, much consideration has been given to the integration of various sensory modalities (somatosensory, visual, auditory, vestibular, visceral) while less attention has been paid to the role that efferent pathways play in shaping the body's inner dimension and representation (Ehrsson, 2007; Lenggenhager et al., 2007; Petkova and Ehrsson, 2008; Guterstam et al., 2015). Patients with LIS may represent an experimental model to better investigate the role of efferent pathways in embodied simulation mechanisms and become a target population for innovative rehabilitative approaches aimed at reducing the percentage of disagreement within the body matrix computational processes. These approaches can include virtual reality and haptic technologies, bio/neuro-feedback strategies and brain/body stimulation paradigms. In patients with LIS, the technology used by Sonoception may be used in the attempt to reduce the mismatch between the efferent (defective) and the afferent (healthy) pathways. For instance, as shown in Figure 1C, vibrotactile transducers may be applied to a physically immobile limb of patients, in order to generate a sensation of arm displacement and to train the self-monitoring of patients. In fact, in healthy subjects, self-monitoring is based on the proper working of an internal forward model: every time that a motor command arises in the motor cortex, this information also reaches the cerebellum where a copy about the command is registered (Wolpert et al., 1995; Ito, 2008). The information transfer is subserved by the corticocerebellar pathways. In this way the cerebellum is able to predict the sensorial consequences of the action resulting from the command and to compare these sensory predictions to the actual sensory feedback received through the spinocebellar tracts (Wolpert et al., 1995; Ito, 2008). If the mismatch between sensory predictions and sensory feedback is little, this confirms that the action is self-generated and leads to an attenuation of the intensity of the sensation associated with the action itself. On the other hand, if the discrepancy between sensory predictions and sensory feedback is high, it is likely that the action is not self-generated and this leads to a relative increase in the intensity of the sensation associated with the stimulus. In patients with LIS the interruption of both corticospinal and corticocerebellar pathways, against the preservation of spinocerebellar pathways, interferes with the proper working of the forward model by producing a continuous mismatch between sensory predictions and sensory feedback. Providing a sensation of arm displacement in these patients may contribute to reduce this mismatch and to restore the functional coupling between motor intentions and sensory feedback. A specific training based on this approach may, in the long term, promote a partial motor recovery, especially when a small proportion of corticospinal fibers had survived the initial injury. This might contribute to improving the well-being of a population of patients whose chances of recovery have always been considered exceedingly small.

## Author contributions

All authors listed have made a substantial, direct and intellectual contribution to the work, and approved it for publication.

### Conflict of interest statement

The authors declare that the research was conducted in the absence of any commercial or financial relationships that could be construed as a potential conflict of interest.
